# Safety, Tolerability, and Pharmacokinetics of the Novel Antiviral Agent Ensitrelvir Fumaric Acid, a SARS-CoV-2 3CL Protease Inhibitor, in Healthy Adults

**DOI:** 10.1128/aac.00632-22

**Published:** 2022-09-12

**Authors:** Ryosuke Shimizu, Takuhiro Sonoyama, Takahiro Fukuhara, Aya Kuwata, Yumiko Matsuo, Ryuji Kubota

**Affiliations:** a Shionogi & Co., Ltd., Clinical Pharmacology & Pharmacokinetics, Osaka, Japan; b Shionogi & Co., Ltd., Medical Science Department, Osaka, Japan; c Shionogi & Co., Ltd., Clinical Research Department, Osaka, Japan

**Keywords:** COVID-19, first-in-human, pharmacokinetics, ensitrelvir, drug-drug interaction, protease inhibitor, antiviral

## Abstract

Ensitrelvir is a novel selective inhibitor of the 3C-like protease of SARS-CoV-2, which is essential for viral replication. This phase 1 study of ensitrelvir assessed its safety, tolerability, and pharmacokinetics of single (part 1, *n* = 50) and multiple (part 2, *n* = 33) ascending oral doses. Effect of food on the pharmacokinetics of ensitrelvir, differences in pharmacokinetics of ensitrelvir between Japanese and white participants, and effect of ensitrelvir on the pharmacokinetics of midazolam (a cytochrome P450 3A [CYP3A] substrate) were also assessed. In part 1, Japanese participants were randomized to placebo or ensitrelvir at doses of 20, 70, 250, 500, 1,000, or 2,000 mg. In part 2, Japanese and white participants were randomized to placebo or once-daily ensitrelvir at loading/maintenance dose 375/125 mg or 750/250 mg for 5 days. Most treatment-related adverse events observed were mild in severity and were resolved without treatment. Plasma exposures showed almost dose proportionality, and geometric mean half-life of ensitrelvir following the single dose was 42.2 to 48.1 h. Food intake reduced *C*_max_ and delayed *T*_max_ of ensitrelvir but did not impact the area under the curve (AUC), suggesting suitability for administration without food restriction. Compared with Japanese participants, plasma exposures were slightly lower for white participants. Ensitrelvir affected the pharmacokinetics of CYP3A substrates because of increase in AUC of midazolam coadministered with ensitrelvir 750/250 mg on day 6. In conclusion, ensitrelvir was well-tolerated and demonstrated favorable pharmacokinetics, including a long half-life, supporting once-daily oral dosing. These results validate further assessments of ensitrelvir in participants with SARS-CoV-2 infection.

## INTRODUCTION

As of April 2022, COVID-19, caused by the severe acute respiratory syndrome coronavirus 2 (SARS-CoV-2), has resulted in 6.2 million deaths worldwide (1). COVID-19 manifests as acute viral exudative pneumonia. In a sizable proportion of patients, the infection can also lead to severe pneumonia and acute respiratory distress syndrome, which is associated with high fatality (2). Considering the high infectivity potential, evolving mutant variants, and life-threatening risks associated with SARS-CoV-2, mass vaccinations and pharmaceutical interventions against COVID-19 are needed as preventive and therapeutic strategies (2, 3). Furthermore, patients may experience a wide range of long-term sequelae of COVID-19 and will benefit from interventions that can halt long-term COVID progression (4).

The unprecedented global burden on the health care through high prevalence and related mortality are caused, to a large extent, by the highly transmissible nature of SARS-CoV-2. SARS-CoV-2 infects cells by binding to the host angiotensin-converting enzyme 2 (ACE2) via the viral spike glycoprotein and releasing the viral RNA into the host cell after uncoating (5–7). Nsp5, or 3C-like protease (3CL protease), is a cysteine protease that is essential for viral replication (8, 9). Preclinical evidence shows that the inhibition of the 3CL protease impairs the formation of enzymes that are essential for viral replication (10). In the viral genome, spike proteins and 3CL proteases are encoded by distinct regions; thus, it may be assumed that the antiviral efficacy of a 3CL protease inhibitor will not be impacted by mutations in the viral spike proteins (11). Furthermore, evidence indicates that the recent variant of concern of SARS-CoV-2, Omicron, includes mutations in the spike protein, which leads to diminished efficacy of Food and Drug Administration (FDA)-approved monoclonal antibodies against this strain (12). Additionally, antiviral treatment options such as remdesivir and molnupiravir have been shown to be efficacious against Omicron (12). Furthermore, recent clinical studies have highlighted the therapeutic potential of nirmatrelvir, which is a peptide-like, covalent, oral 3CL protease inhibitor (13, 14). These findings posit the 3CL protease molecule as a potentially stable target for antiviral agents. However, the elimination of nirmatrelvir in humans is rapid due to low metabolic stability (13, 14), and nirmatrelvir requires the coadministration of ritonavir as a pharmacokinetic booster to maintain the target exposure. Consequently, this leads to a limitation in the use of nirmatrelvir due to potential drug-drug interactions (DDIs) with ritonavir.

In a recent study, we described the discovery of S-217622 or ensitrelvir, an oral SARS-CoV-2 3CL protease inhibitor for potential use in the treatment of COVID-19 (15). Preclinical studies have demonstrated antiviral activity of ensitrelvir against a broad spectrum of SARS-CoV-2 variants, including the Omicron strain, and coronavirus families and have exhibited its favorable drug metabolism and pharmacokinetic profiles (15). Furthermore, ensitrelvir showed dose-dependent antiviral activity in BALB/cAJcl mice, indicating its potential in the treatment of COVID-19 (15).

The aim of this phase 1 study was to assess the safety, tolerability, and pharmacokinetics of single and multiple doses of ensitrelvir by suspension formulation in healthy participants. The effect of food, the comparison of pharmacokinetics of ensitrelvir in healthy Japanese and white participants, and DDI for midazolam, a cytochrome P450 3A (CYP3A) substrate, were also evaluated. This manuscript reports the findings from a part of the ongoing study. Data from additional cohorts, including Japanese females, and the DDI with dexamethasone or prednisolone in Japanese adult males and others will be discussed separately.

## RESULTS

### Study participants and baseline demographics.

A total of 50 healthy adult males participated in part 1 (cohorts A, B, C, D, E, and J; single-dose part) and 33 healthy adult males participated in part 2 (cohorts F, G, and H; multiple-dose part) of the study. Single doses of 20 to 2,000 mg ensitrelvir or placebo were administered in part 1. The dosing schedule of ensitrelvir in part 2 was a loading dose of 375 mg on day 1 followed by a maintenance dose of 125 mg from days 2 to 5 (375/125 mg), or a loading dose of 750 mg on day 1 followed by a maintenance dose of 250 mg from days 2 to 5 and day 6 with midazolam (750/250 mg). Corresponding placebo were administered on day 1 to 5 or day 6. In part 1, the mean (SD) age ranged from 24.0 (4.2) to 32.8 (9.9) years and the mean (SD) body mass index (BMI) ranged from 22.38 (0.76) to 24.75 (2.01) kg/m^2^ across all cohorts (Table S1). In part 2, across all cohorts, the mean (SD) age ranged from 29.4 (7.3) to 36.0 (6.9) years, and the mean (SD) BMI ranged from 21.10 (1.35) to 24.40 (2.00) kg/m^2^ (Table S2).

### Pharmacokinetics.

**FIG 1:**
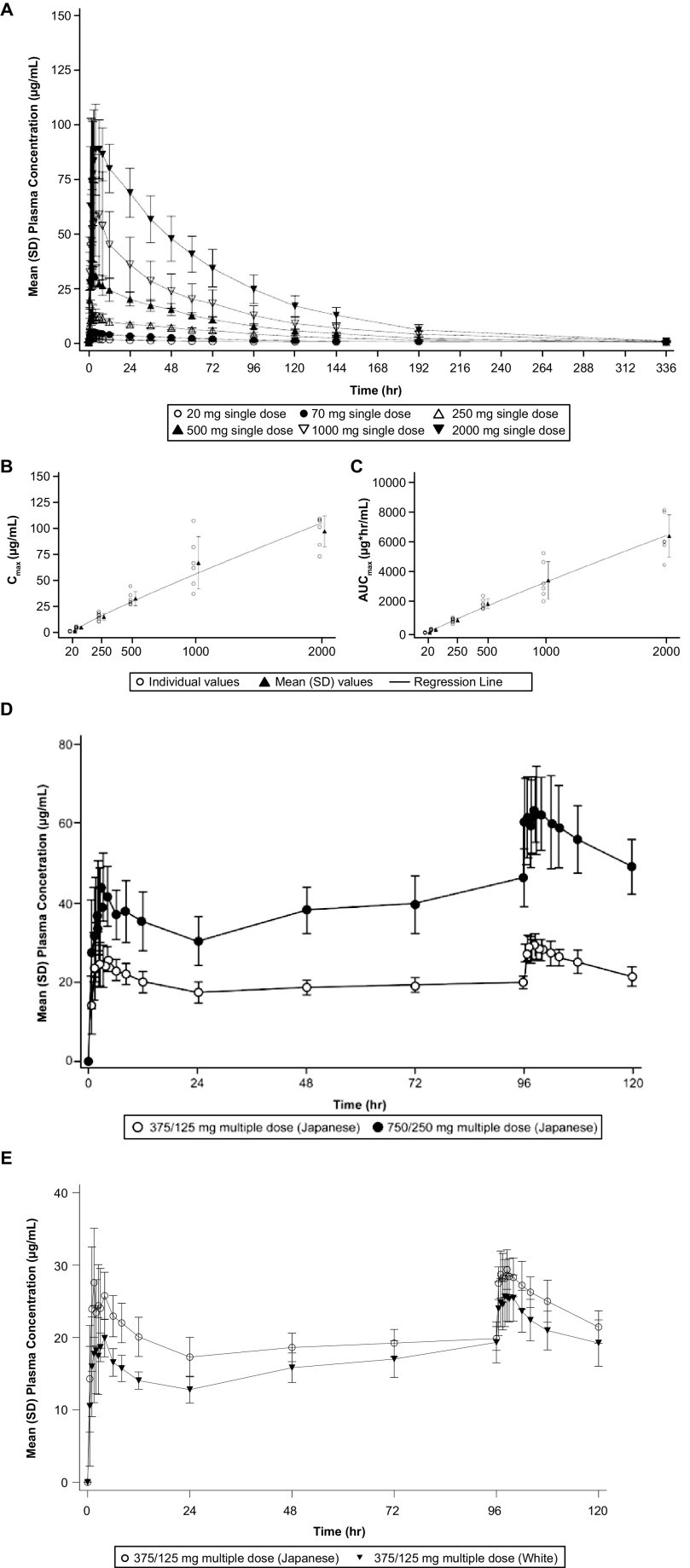
Pharmacokinetic profile of single- and multiple-dose administration of ensitrelvir and dose-proportionality following single-dose administration of ensitrelvir. Mean (SD) plasma concentration profile of ensitrelvir following administration of ensitrelvir: (A) single-dose (dose range, 20 to 2,000 mg; cohorts A–E and J) in the fasted state in Japanese healthy adult male participants (part 1); plots of (B) *C*_max_ and (C) AUC_0-inf_ of ensitrelvir versus dose after single-dose administration of ensitrelvir (dose range, 20–2,000 mg; cohorts A to E and J) in the fasted state in Japanese healthy adult male participants (part 1); (D) multiple-dose in the fasted state in Japanese healthy male participants (cohorts F and G); (E) multiple-dose in the fasted state in Japanese and white healthy adult male participants (cohorts F and H).

The plasma concentration-time profiles of ensitrelvir in the single and multiple ascending-dose administrations in the fasted state are shown in Fig. 1. Plasma and urine ensitrelvir pharmacokinetic parameters are shown in Table 1 and 2.

**TABLE 1:** Pharmacokinetic parameters for single-dose administration of ensitrelvir (part 1; cohorts A–E, and J) in the fasted state^*a*^

Parameters	Ensitrelvir (cohort/dose)					
	A/20 mg	B/70 mg	C/250 mg	D/500 mg	E/1000 mg	J/2000 mg
*n*	6	6	8	6	6	6
*C*_max_ (μg/mL)	1.70 (15.0)	5.20 (18.5)	15.2 (23.6)	32.6 (19.0)	63.8 (39.1)	96.9 (16.5)
*T*_max_ (h)	2.50 (1.00, 4.00)	1.50 (1.00, 4.00)	2.50 (1.00, 12.00)	2.00 (1.00, 4.00)	2.75 (1.00, 6.00)	4.00 (1.50, 8.00)
AUC_0-last_ (μg·h/mL)	82.00 (19.5)	289.1 (15.4)	906.8 (15.8)	1975 (15.9)	3,341 (35.2)	6,311 (22.0)
AUC_0-inf_ (μg·h/mL)	91.44 (24.3)	291.0 (15.7)	913.7 (16.2)	1987 (16.1)	3,370 (35.5)	6,346 (22.2)
t_1/2,z_ (h)	42.6 (18.6)	45.7 (11.9)	43.1 (20.2)	42.2 (14.6)	48.1 (11.3)	43.1 (15.6)
MRT (h)	61.8 (18.7)	67.3 (13.4)	73.4 (12.7)	68.2 (9.0)	70.3 (9.3)	67.4 (8.4)
CL/F (L/h)	0.219 (24.3)	0.241 (15.7)	0.274 (16.2)	0.252 (16.1)	0.297 (35.5)	0.315 (22.2)
V_z_/F (L)	13.5 (10.2)	15.9 (9.0)	17.0 (8.8)	15.3 (13.5)	20.6 (26.2)	19.6 (21.7)
Feu_0-144_ (%)	12.9 (14.6)	14.7 (27.4)	16.0 (16.6)	21.8 (15.4)	19.4 (29.5)	NA
CL_R_ (L/h)	0.0314 (25.0)	0.0401 (34.9)	0.0507 (21.2)	0.0624 (15.4)	0.0668 (24.7)	NA

a Geometric means (percentage coefficient of variation) are presented, except for *T*_max_, for which medians (minimum, maximum) are presented. NA, not applicable.

**TABLE 2:** Pharmacokinetic parameters for multiple-dose administration of ensitrelvir (part 2; cohorts F, G, and H) in the fasted state^*a*^

Parameter	Ensitrelvir (cohort/dose)					
	F/375/125 mg (Japanese)		G/750/250 mg (Japanese)		H/375/125 mg (white)	
	Day 1	Day 5	Day 1	Day 5	Day 1	Day 5
*n*	8	8	8	7*	8	8
*C*_max_ (μg/mL)	29.5 (18.6)	30.4 (8.0)	44.8 (21.4)	66.3 (16.0)	22.7 (10.9)	26.3 (15.3)
*T*_max_ (h)	1.50 (1.50, 4.00)	2.00 (1.00, 6.00)	3.00 (2.00, 3.00)	2.50 (0.50, 6.00)	1.75 (0.50, 4.00)	1.50 (0.50, 4.00)
AUC_0-τ_ (μg·h/mL)	484.5 (13.6)	597.4 (10.2)	818.4 (20.8)	1,337 (15.0)	350.8 (10.2)	516.5 (14.6)

^*a*^Geometric means (percentage coefficient of variation) are presented, except for *T*_max_, for which medians (minimum, maximum) are presented. 375/125 mg, multiple once-daily doses with 375 mg as the loading dose on day 1 and 125 mg as the maintenance dose on days 2 to 5; 725/250 mg, multiple once-daily doses with 750 mg as the loading dose on day 1 and 250 mg as the maintenance dose on days 2 to 6. *, one patient discontinued due to adverse events (rash, feeling hot, and headache) on day 2, but eventually recovered.

**(i) Single-dose pharmacokinetics of ensitrelvir.** Mean (SD) plasma concentration profiles of ensitrelvir following single-dose administration (dose range, 20 to 2,000 mg; cohorts A to E and J) in the fasted state in Japanese healthy adult participants are presented in Fig. 1A. Following single-dose administration at 20 to 2,000 mg in the fasted state to Japanese healthy adult participants, the median time to maximum plasma concentration (*T*_max_) of ensitrelvir ranged from 1.5 to 4 h and the geometric mean terminal elimination half-life (t_1/2,z_) of ensitrelvir was 42.2 to 48.1 h. Plasma parameters after single-dose administration versus ensitrelvir dose are shown in Fig. 1B and C, with plots of maximum plasma concentration (*C*_max_) and area under the concentration-time curve extrapolated to infinity (AUC_0-inf_), respectively. According to a power model for dose proportionality of *C*_max_ and AUC_0-inf_ across the dose range from 20 to 2,000 mg, the slopes (95% confidence intervals [CIs]) were 0.899 (0.850–0.948) for *C*_max_ and 0.926 (0.880–0.972) for AUC_0-inf_, suggesting that plasma exposures of ensitrelvir increased in an almost dose-proportional manner across the dose ranges after single-dose administration, although the upper limit of 95% CIs were less than 1. Urinary recovery of ensitrelvir was observed and the geometric mean of the fraction of dose excreted in urine from time zero to 144 h (Feu_0-144_) of ensitrelvir ranged from 12.9% to 21.8% across dose groups 20 to 1,000 mg.

The 90% CIs of the geometric least-squares (GLS) mean ratio for t_1/2,z_ contained 1 for the groups that received 20 to 2,000 mg of ensitrelvir, suggesting dose independency of t_1/2,z_ across the dose range. The point estimates of the GLS mean ratios for mean residence time (MRT) were close to 1, and the 90% CIs of the GLS mean ratios almost contained 1 for groups that received 20 to 2,000 mg of ensitrelvir. Although the lower limits of 90% CIs of the GLS mean ratios of apparent total clearance (CL/F) and apparent volume of distribution in the terminal elimination phase (V_z_/F) exceeded 1 in some dose group combinations, the GLS mean ratios for CL/F were similar in each dose combination, and no clear relationships between V_z_/F with dose were observed. These results suggested dose independency of these pharmacokinetic parameters of ensitrelvir in the fasted state.

**(ii) Multiple-dose pharmacokinetics of ensitrelvir.** Figure 1D displays plasma concentration-time profiles following multiple-dose administrations of ensitrelvir, given at 375/125 mg for 5 days or 750/250 mg for 5 days in the fasted state, to healthy Japanese adult participants (cohorts F and G). The *C*_max_ and area under the concentration-time curve overdosing interval τ (AUC_0-τ_) of ensitrelvir slightly increased following multiple-dose administration of ensitrelvir. The GLS mean ratios (90% CIs) (cohort G [750/250 mg] versus F [375/125 mg]) for *C*_max_ and AUC_0-τ_ of ensitrelvir on day 1 were 1.5185 (1.2747–1.8089) and 1.6893 (1.4492–1.9692), respectively, and those on day 5 were 2.1781 (1.9460–2.4379) and 2.2387 (1.9942–2.5132), respectively, suggesting that the *C*_max_ and AUC of ensitrelvir increased in an almost dose-proportional manner across the dose ranges after multiple-dose administration.

The plasma concentration profiles of ensitrelvir from days 1 to 5 following multiple doses given to Japanese and white healthy adult participants are presented in Fig. 1E. Comparisons of pharmacokinetic parameters of ensitrelvir between Japanese and white healthy adult participants (375/125 mg, Japanese versus 375/125 mg, white; cohort F versus H) following multiple-dose administration of ensitrelvir revealed that the *C*_max_ and AUC_0-τ_ in white participants were slightly lower than those in Japanese participants on both days 1 and 5 (Fig. 1E). The GLS mean ratios (90% CIs) of *C*_max_ (white/Japanese) of ensitrelvir on day 1 and say 5 were 0.7699 (0.6738–0.8796) and 0.8651 (0.7774–0.9627), respectively. Corresponding GLS mean ratios (90% CIs) of AUC_0-τ_ of ensitrelvir were 0.7241 (0.6517–0.8046) and 0.8645 (0.7740–0.9656). The 90% CIs of the ratios for *C*_max_ and AUC_0-τ_ of ensitrelvir on day 1 and day 5 did not include 1, and the lower limits of those 90% CIs were below the lower limit of the equivalence range (0.8000).

**FIG 2:**
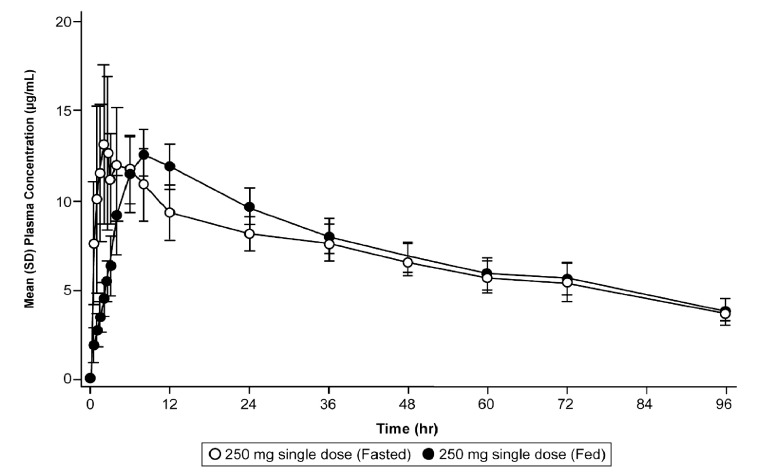
Effect of food on the pharmacokinetics of ensitrelvir. Mean (SD) plasma concentration profile of ensitrelvir following single-dose administration of ensitrelvir 250 mg in the fasted and fed states in Japanese healthy adult participants.

**(iii) Effect of food on the pharmacokinetics of ensitrelvir.** Fig. 2 shows the plasma concentration-time profiles of ensitrelvir following single-dose administration in the fasted and fed state for cohort C. The corresponding results of statistical analysis for plasma ensitrelvir pharmacokinetic parameters are presented in Table 3. Food intake reduced the *C*_max_ of ensitrelvir by 15% and delayed the *T*_max_ of ensitrelvir from 2.5 h in the fasted state to 8 h in the fed state. However, no impact of ensitrelvir was noted in AUC.

**TABLE 3:** Statistical analysis of the effect of food on the pharmacokinetics of ensitrelvir following single-dose 250-mg administration in the fasted and fed states (part 1)

	Cohort C/250 mg single dose				GLS mean (90% CI) ratio (fed/fasted)
Parameter	Fasted		Fed		
	*n*	GLS mean	*n*	GLS mean	GLS mean (90% CI) ratio
*C*_max_ (μg/mL)	8	15.2	8	13.0	0.8508 (0.7507/0.9644)
AUC_0-last_ (μg·h/mL)	8	906.8	8	949.4	1.0470 (1.0061/1.0895)
AUC_0-inf_ (μg·h/mL)	8	913.7	8	955.7	1.0460 (1.0047/1.0891)
t_1/2,z_	8	43.1	8	40.5	0.9385 (0.8893/0.9905)

**FIG 3:**
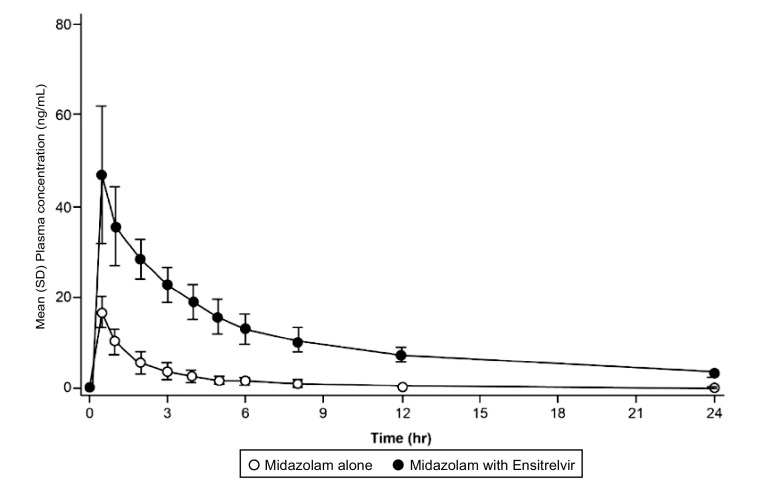
Drug-drug interaction. Mean (SD) plasma concentration profile of midazolam following single-dose administration of midazolam alone and coadministered with ensitrelvir on day 6.

**(iv) DDI of ensitrelvir with midazolam.** Fig. 3 shows the mean (SD) plasma midazolam concentration profile (cohort G) following single-dose administration of midazolam alone and coadministered with ensitrelvir on day 6 (750/250 mg for 6 days once daily). The *C*_max_, area under the concentration-time curve to the last measurable concentration (AUC_0-last_), and AUC_0-inf_ of midazolam coadministered with ensitrelvir increased 2.78-, 7.23-, and 8.80-fold, respectively, compared to the corresponding values for midazolam alone (Table 4). The increase in AUC_0-inf_ of midazolam upon coadministration with ensitrelvir suggested that ensitrelvir 750/250 mg acts as a strong CYP3A inhibitor.

**TABLE 4:** Statistical analysis of the effect of ensitrelvir on the pharmacokinetics of midazolam following single-dose administration of midazolam (2 mg) alone and coadministered with ensitrelvir (750/250 mg)^*a*^

Parameter	Midazolam alone		Midazolam with ensitrelvir		GLS mean (90% CI) ratio (midazolam with ensitrelvir/midazolam alone)
	*n*	GLS mean	*n*	GLS mean	GLS mean (90% CI) ratio
*C*_max_ (ng/mL)	8	16.2	7	44.9	2.7763 (2.3341/3.3022)
AUC_0-last_ (ng·h/mL)	8	34.70	7	250.8	7.2293 (5.5817/9.3632)
AUC_0-inf_ (ng·h/mL)	8	35.69	5	314.1	8.8002 (6.7061/11.5484)
λ_z_ (1/h)	8	0.1590	7	0.0759	0.4772 (0.4439.0.5131)
t_1/2,z_ (h)	8	4.36	7	9.13	2.0954 (1.9490/2.2529)
MRT (h)	8	4.11	5	11.8	2.8734 (2.5380/3.2530)

*^a^*725/250 mg, multiple once-daily doses with 750 mg as the loading dose on day 1 and 250 mg as the maintenance dose on days 2 to 6.

**(v) Safety and tolerability**. Tables 5 and 6 show the major treatment-emergent adverse events (TEAEs) observed for ensitrelvir (4A) single-dose and (4B) multiple-dose administrations. TEAEs observed in ≥2 participants in the ensitrelvir group were decreased high-density lipoprotein cholesterol (HDL-C) (6/6 both in cohorts E and J; 7/8 each in cohorts F, G, and H), diarrhea (4/8 each in cohorts F, G, and H), headache (4/8 in cohort G and 2/8 in cohort H), and somnolence (3/8 in cohort G), which was observed after administration of midazolam. TEAEs in cohort G were more frequent than the other cohorts. This may reflect the higher dose but is also complicated by the concomitant administration of midazolam (16). Most TEAEs observed were mild in severity and were resolved without treatment. Furthermore, the decrease in HDL-C levels was transient and generally reverted to the baseline values and was associated with a decrease in triglyceride levels (Fig. S2 and Table S3).

**TABLE 5:** Treatment-emergent adverse events following single-dose administration of ensitrelvir (20–2,000 mg in cohorts A to E and J) in Japanese healthy adult male participants (part 1; cohorts A–E, and J) in the fasted state^*a*^

System organ class preferred term^*b*^	Ensitrelvir												Placebo	
	Cohort A 20 mg		Cohort B 70 mg		Cohort C (fasted) 250 mg		Cohort D 500 mg		Cohort E 1,000 mg		Cohort J 2,000 mg		(Fasted)^*c*^	
	*n* = 6		*n* = 6		*n* = 8		*n* = 6		*n* = 6		*n* = 6		*n* = 12	
	*n* (%)	Events	*n* (%)	Events	*n* (%)	Events	*n* (%)	Events	*n* (%)	Events	*n* (%)	Events	*n* (%)	Events
Participants with any TEAE	0	0	2 (33.3)	2	0	0	0	0	6 (100)	6	6 (100)	11	0	0
Nervous system disorders	0	0	1 (16.7)	1	0	0	0	0	0	0	1 (16.7)	1	0	0
Headache	0	0	1 (16.7)	1	0	0	0	0	0	0	1 (16.7)	1	0	0
Gastrointestinal disorders	0	0	1 (16.7)	1	0	0	0	0	0	0	1 (16.7)	3	0	0
Abdominal pain	0	0	1 (16.7)	1	0	0	0	0	0	0	0	0	0	0
Nausea	0	0	0	0	0	0	0	0	0	0	1 (16.7)	1	0	0
Vomiting	0	0	0	0	0	0	0	0	0	0	1 (16.7)	1	0	0
Feces soft	0	0	0	0	0	0	0	0	0	0	1 (16.7)	1	0	0
General disorders and administration site conditions	0	0	0	0	0	0	0	0	0	0	1 (16.7)	1	0	0
Chills	0	0	0	0	0	0	0	0	0	0	1 (16.7)	1	0	0
Investigations	0	0	0	0	0	0	0	0	6 (100)	6	6 (100)	6	0	0
High density lipoprotein cholesterol decreased	0	0	0	0	0	0	0	0	6 (100)	6	6 (100)	6	0	0

a All adverse events were mild.

b System organ class and preferred term of MedDRA ver. Event, number of events; 24.0; *n*, number of participants.

c Placebo (fasted state) includes all patients given placebo in cohorts A, B, C (fasted), D, E, and J; TEAE, treatment-emergent adverse event; event, number of events; N, number of participants.

**TABLE 6:** Treatment-emergent adverse events following multiple-dose administration of ensitrelvir suspension in Japanese healthy adult male participants (cohorts F and G) and white healthy male participants (cohort H) (part 2)^*a*^

System organ class preferred term^*b*^	Ensitrelvir						Placebo					
	Cohort F 375/125 mg		Cohort G 750/250 mg + midazolam		Cohort H 125 mg		Cohort F 125 mg		Cohort G 750/250 mg + midazolam		Cohort H 375/125 mg	
	*n* = 8		*n* = 8		*n* = 8		*n* = 3		*n* = 3		*n* = 3	
	*n* (%)	Events	*n* (%)	Events	*n* (%)	Events	*n* (%)	Events	*n* (%)	Events	*n* (%)	Events
Participants with any TEAE	7 (87.5)	12	8 (100)	33	8 (100)	19	0	0	1 (33.3)	1	1 (33.3)	1
Nervous system disorders	0	0	5 (62.5)	11	2 (25.0)	4	0	0	0	0	0	0
Headache	0	0	4 (50.0)	6	2 (25.0)	4	0	0	0	0	0	0
Somnolence	0	0	3 (37.5)	4	0	0	0	0	0	0	0	0
Dizziness	0	0	1 (12.5)	1	0	0	0	0	0	0	0	0
Respiratory, thoracic, and mediastinal disorders	0	0	0	0	1 (12.5)	1	0	0	0	0	0	0
Oropharyngeal pain	0	0	0	0	1 (12.5)	1	0	0	0	0	0	0
Gastrointestinal disorders	4 (50.0)	5	4 (50.0)	8	4 (50.0)	7	0	0	1 (33.3)	1	1 (33.3)	1
Diarrhea	4 (50.0)	5	4 (50.0)	6	4 50.0)	6	0	0	1 (33.3)	1	1 (33.3)	1
Abdominal pain	0	0	0	0	1 (12.5)	1	0	0	0	0	0	0
Nausea	0	0	1 (12.5)	1	0	0	0	0	0	0	0	0
Vomiting	0	0	1 (12.5)	1	0	0	0	0	0	0	0	0
Skin and subcutaneous tissue disorders	0	0	1 (12.5)	1	0	0	0	0	0	0	0	0
Rash	0	0	1 (12.5)	1	0	0	0	0	0	0	0	0
General disorders and administration site conditions	0	0	2 (25.0)	2	0	0	0	0	0	0	0	0
Feeling hot	0	0	1 (12.5)	1	0	0	0	0	0	0	0	0
Pyrexia	0	0	1 (12.5)	1	0	0	0	0	0	0	0	0
Investigations	7 (87.5)	7	7 (87.5)	11	7 (87.5)	7	0	0	0	0	0	0
High-density lipoprotein cholesterol decreased	7 (87.5)	7	7 (87.5)	7	7 (87.5)	7	0	0	0	0	0	0
Blood triglyceride increased	0	0	2 (25.0)	2	0	0	0	0	0	0	0	0
Blood creatine phosphate increased	0	0	1 (12.5)	1	0	0	0	0	0	0	0	0
C-reactive protein increased	0	0	1 (12.5)	1	0	0	0	0	0	0	0	0

a 375/125 mg, multiple once-daily doses with 375 mg as the loading dose on day 1 and 125 mg as the maintenance dose on days 2 to 5; 725/250 mg, multiple once-daily doses with 750 mg as the loading dose on day 1 and 250 mg as the maintenance dose on days 2 to 6. Rash (*n* = 1) and nausea (*n* = 1) in cohort G were moderate, and all the other AEs were mild.

b System organ class and preferred term of MedDRA ver. 24.0.

## DISCUSSION

This study describes the results of the first-in-human assessment of ensitrelvir, a potential agent for use in the treatment of COVID-19. Here, we report the findings of a phase 1 study assessing the safety, tolerability, and pharmacokinetics of single and multiple ascending doses of ensitrelvir.

In this report, ensitrelvir showed dose-proportional pharmacokinetics in a wide dose range from 20 to 2,000 mg following single-dose administration. The elimination half-life of ensitrelvir was long, which enabled once-daily administration. Furthermore, ensitrelvir could be administered without food restriction because food intake did not affect the AUC of ensitrelvir, although the rate of absorption became slower with intake of high-fat, high-calorie food. Thus, ensitrelvir showed the preferable pharmacokinetic characteristics as an oral therapeutic drug. In the present study, only 12.9% to 21.8% of ensitrelvir was recovered in the urine across dose groups ranging from 20 to 1,000 mg, suggesting partial contribution of urinary elimination to the elimination of ensitrelvir in humans.

Antiviral drug therapy is most beneficial when started in the early phase of SARS-CoV-2 infection (17). Thus, the initial dose was set to achieve the plasma drug concentration necessary for viral suppression on the first day of intervention (day 1) based on the single-dose ascending part. Then, the daily maintenance dose was set as one-third of the initial dose to keep a constant exposure based on the accumulated pharmacokinetics profile. In multiple-dose administration, constant exposures were obtained from the initial to the last day of administration by the set dose regimens. Comparison of pharmacokinetic parameters of ensitrelvir between Japanese and white participants following multiple-dose administration of ensitrelvir revealed that the *C*_max_ and AUC_0-τ_ in white participants were slightly lower than those in Japanese participants. The difference in the pharmacokinetic profiles between Japanese and white participants may be at least partially attributed to the differences in body weight because the Japanese participants (mean weight, 66.58 kg) had lower body weight than white participants (mean weight, 74.68 kg). The influential factors for pharmacokinetics of ensitrelvir will be explored and reported as separate research.

The FDA recommends the use of midazolam as a CYP3A substrate to evaluate the potential DDI of investigational drugs with CYP3A inhibitors in clinical studies (18, 19). In the current study, the AUC of midazolam was increased up to 8.80-fold with 750/250 mg ensitrelvir, suggesting that ensitrelvir acts as a strong CYP3A inhibitor following multiple doses (750/250 mg) for 5 days and additional coadministration with midazolam on day 6. Further investigations are needed to examine the impact of a lower dosing regimen (375/125 mg) as a clinical dose for ensitrelvir by using a modeling and simulation approach based on the clinical DDI results.

As per our safety results, ensitrelvir was well tolerated across all doses tested and no major safety concerns were observed. The most common TEAEs for ensitrelvir, observed in ≥2 participants, were decreased high-density lipoprotein cholesterol (HDL-C), diarrhea, and headache (Table 5 and 6). In addition, somnolence was reported after administration of midazolam. All TEAEs observed for both single- and multiple-dose administrations of ensitrelvir were mild and resolved spontaneously. Decrease in HDL-C was observed in both single-dose and multiple-dose administration, and the mechanism of this change remains unknown. However, this change was transient and HDL-C level generally reverted to the baseline value, and there were no other notable changes in hepatic markers or clinical signs associated with the increased HDL-C. Thus, the transient reduction in HDL-C reported in this study may not be related to adverse clinical implications. Furthermore, it is important to note that the mechanisms of a decrease of HDL-C are currently unclear and further investigation on this is warranted.

There is a need for antiviral drugs that can quickly reduce the viral load in the body, prevent the development of clinical symptoms such as fever or respiratory symptoms, and avoid severe exacerbations. Data presented in this first-in-human study indicate that ensitrelvir was generally safe and well-tolerated in healthy participants, and the pharmacokinetics evaluations highlighted the potential for once-daily administration. These findings support further clinical development of ensitrelvir in participants with COVID-19 and provide a rationale for the selection of doses for subsequent studies. Importantly, this study quantitatively describes that ensitrelvir dosing at 375/125 mg is expected to achieve the target exposure levels associated with expected antiviral activity established based on nonclinical results (15). Furthermore, the oral route of administration of once-daily ensitrelvir renders it convenient for administration in the outpatient setup.

In conclusion, ensitrelvir was well-tolerated and demonstrated favorable pharmacokinetics, including a long half-life, supporting once-daily oral dosing. In this regard, ensitrelvir 375/125 mg and 750/250 mg have now been advanced for a phase 2/3 study, which is ongoing.

## MATERIALS AND METHODS

### Study design.

A phase 1, multicenter, two-part, double-blinded, randomized, placebo-controlled study is being conducted from July 22, 2021. We hereby present the results of parts of this study that have been completed by December 27, 2021. Healthy adult male participants were randomized to receive single-dose (part 1) and multiple doses (part 2) of orally administered placebo or ensitrelvir fumaric acid (ensitrelvir) in a suspension formulation (Fig. S1 and Tables S3A and 3B). Participants were randomized 3:1 to ensitrelvir and placebo for part 1 (cohorts A, B, D, E, and J); 4:1 for cohort C; and 8:3 for part 2 (cohorts F, G, and H).

In part 1, Japanese participants were randomized to receive placebo or single dose of ensitrelvir (20, 70, 250, 500, 1,000, and 2,000 mg given to cohorts A, B, C, D, E, and J, respectively), suspended in 0.5% methylcellulose, in the fasted state. Additionally, in cohort C, the effect of food on the pharmacokinetics of ensitrelvir was investigated in a 2-group, 2-period crossover design by administering placebo or ensitrelvir (250 mg) in the fasted or fed (high-fat/high-calorie) state. In part 2, Japanese participants were randomized to receive placebo or multiple doses of ensitrelvir, suspended in 0.5% methylcellulose. The multiple-dose regimens were as follows: once-daily doses of ensitrelvir 375 mg on day 1 and 125 mg on days 2 to 5 (cohort F), or 750 mg on day 1 and 250 mg on days 2 to 6 (cohort G) in the fasted state. White participants were randomized to receive placebo or multiple doses of ensitrelvir. The multiple-dose regimens were as follows: once-daily doses of ensitrelvir 375 mg on day 1 and 125 mg on days 2 to 5 (cohort H). Each cohort included 11 participants. In cohort G, the effect of ensitrelvir on the pharmacokinetics of midazolam (syrup) was evaluated in an open label self-controlled crossover design. Midazolam 2 mg alone was administered 2 days prior to initiating ensitrelvir administration (on day −2) and midazolam was coadministered on day 6 with placebo or ensitrelvir in the fasted state to participants following once-daily doses for 5 days of placebo (days 1 to 5) or ensitrelvir (750 mg on day 1 and 250 mg on days 2–5).

### Ethical compliance.

The study (trial identification no. jRCT2031210202) was conducted in compliance with the protocol approved by Institutional Review Board, the Declaration of Helsinki (WMA) and Council for International Organizations of Medical Sciences International Ethical Guidelines (CIOMS), the International Council for Harmonisation of Technical Requirements for Pharmaceuticals for Human Use Good Clinical Practice Guidelines [ICH], and other applicable laws and regulations. It was also approved by the concerned Institutional Review Board. All participants gave their written informed consent for participation in the study (20–22).

### Bioanalytical procedure.

Plasma and urine concentrations of ensitrelvir were determined by liquid chromatography with tandem mass spectrometry (LC/MS/MS) following protein precipitation with acetonitrile. LC/MS/MS analysis was performed using TSQ Altis (Thermo Fisher Scientific, Waltham, MA). Plasma concentrations of midazolam were determined by LC/MS/MS following solid-phase extraction. LC/MS/MS analysis was performed using SCIEX Triple Quad 6500 (Sciex, Framingham, MA). The plasma and urine concentrations of ensitrelvir were determined at Shionogi & Co., Ltd., and plasma concentrations of midazolam were determined at Shin Nippon Biomedical Laboratories, Ltd.

### Pharmacokinetic assessments.

Blood and urine samples were collected and analyzed at several time points to determine the concentration of ensitrelvir and midazolam. For single-dose administration, blood samples were collected predose (0 h) and 0.5, 1, 1.5, 2, 2.5, 3, 4, 6, 8, 12, 24, 36, 48, 60, 72, 96, 120, and 144 h postdose on day 1 at 20 mg (cohort A); predose (0 h) and 0.5, 1, 1.5, 2, 2.5, 3, 4, 6, 8, 12, 24, 36, 48, 60, 72, 96, 120, 144, 192, and 336 h postdose on day 1 at 70, 500, 1,000, and 2,000 mg (cohorts B, D, E, and J); and predose (0 h) and 0.5, 1, 1.5, 2, 2.5, 3, 4, 6, 8, 12, 24, 36, 48, 60, 72, 96, 120, and 144, 192, and 336 h postdose on days 1 and 15 at 250 mg (cohort C). Urine samples were collected at 0 to 24, 24 to 48, 48 to 72, 72 to 96, 96 to 120, and 120 to 144 h postdose on day 1 at 20, 70, 500, and 1,000 mg (cohorts A, B, D, and E); and 0 to 24, 24 to 48, 48 to 72, 72 to 96, 96 to 120, and 120 to 144 h postdose on days 1 and 15 at 250 mg (cohort C). For multiple-dose administration, blood samples were collected predose (0 h) and 0.5, 1, 1.5, 2, 2.5, 3, 4, 6, 8, and 12 h postdose on day 1; predose (0 h) on days 2 to 4; and predose (0 h) and 0.5, 1, 1.5, 2, 2.5, 3, 4, 6, 8, 12, and 24 h postdose on day 5 to evaluate the pharmacokinetics of ensitrelvir. Blood samples were also collected predose (0 h) and 0.5, 1, 2, 3, 4, 5, 6, 8, 12, and 24 h postdose on days 2 and 6 to evaluate the pharmacokinetics of midazolam.

Pharmacokinetic parameters were estimated based on the plasma concentration time data for ensitrelvir and midazolam by noncompartmental analysis (Phoenix WinNonlin version 6.2.1, Certara USA Inc., Princeton, NJ, USA). Dose proportionality assessments included the *C*_max_, area under the plasma concentration-time curve (AUC_0-last_, AUC_0-inf_, and AUC_0-τ_), and dose independency included t_1/2,z_, terminal elimination rate constant (λ_z_), MRT, CL/F, and V_z_/F. Urine pharmacokinetic parameters for ensitrelvir were calculated based on the urine concentrations of ensitrelvir and urine volume with noncompartmental analysis. Feu_0-144_ was calculated after single-dose administration. Additionally, renal clearance (CL_R_) was calculated using the cumulative amount of the drug excreted in urine and AUC in plasma for ensitrelvir. For calculation of pharmacokinetic parameters, plasma concentrations below the lower limit of quantification (BLQ) before the occurrence of the first measurable concentration was treated as zero, and BLQ after the first occurrence of the measurable concentration was treated as missing. Urine concentrations below the lower limit of quantification were treated as zero for estimation of cumulative amount of the drug excreted in urine.

### Safety and tolerability analyses.

The nature, frequency, and severity of TEAEs were evaluated and recorded.

### Statistical analyses.

The descriptive statistical analyses for pharmacokinetic parameters were performed using SAS (version 9.4). Dose proportionality for *C*_max_ and AUC was examined by using a power model for single-dose administration and an analysis of variance (ANOVA) for multiple-dose administration. Dose independency for pharmacokinetic parameters after single-dose administration, the effect of food on the pharmacokinetics of ensitrelvir, the ratio of exposures between the first dose and last dose following multiple-dose administration of ensitrelvir, and the comparison of pharmacokinetic parameters between Japanese and white healthy adult participants were examined by using ANOVA. Dose independency was concluded based on the point estimates and 90% CIs of the ANOVA model and visual inspection of the corresponding plots. Regarding the evaluation of DDI with midazolam, the effect of multiple-dose administration of ensitrelvir on the pharmacokinetics of midazolam was examined by using ANOVA. If the 90% CIs for the ratios of *C*_max_, AUC_0-last_, and AUC_0-inf_ when coadministered with ensitrelvir on day 6 to those of midazolam administered alone were completely contained within the range of 0.8000 to 1.2500, then it was concluded that multiple-dose administration of ensitrelvir did not affect the pharmacokinetics of midazolam.

The effect of food was concluded based on the point estimates and 90% CIs of the ANOVA model and the comparison of plasma concentration profiles and pharmacokinetic parameters between the fasted and fed state.
