# Supplementary material for: Safety, Tolerability, and Pharmacokinetics of the Novel Antiviral Agent Ensitrelvir Fumaric Acid, a SARS-CoV-2 3CL Protease Inhibitor, in Healthy Adults
Source: Antimicrob Agents Chemother. 2022 Sep 12;66(10):e00632-22. doi: 10.1128/aac.00632-22 (PMC9578392; doi:10.1128/aac.00632-22)
Supplement: Supplemental file 1 — Supplemental material. Download aac.00632-22-s0001.pdf, PDF file, 0.5 MB [file aac.00632-22-s0001.pdf]

Supplementary Material

Supplementary Figure S1: Study design

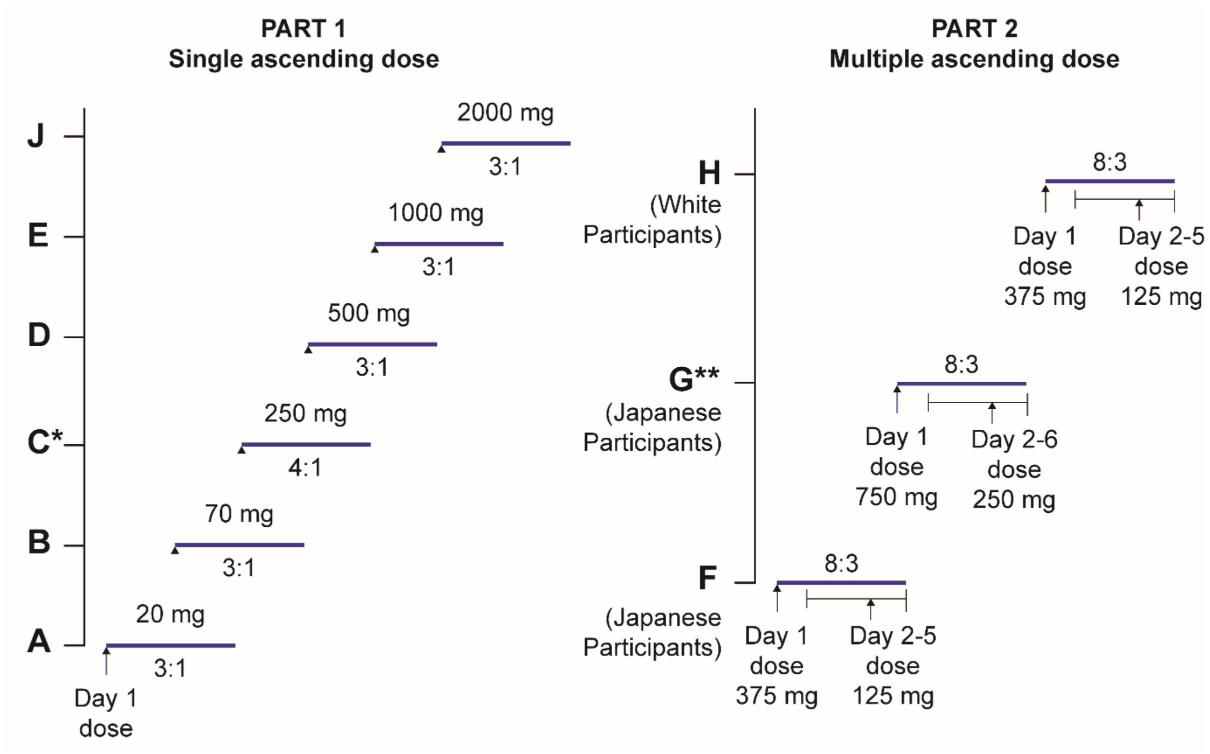

*****In Part, Cohort C also involved the evaluation of the effect of food on the pharmacokinetics of ensitrelvir. In Part 2, Cohort G also involved the evaluation of the impact of ensitrelvir on the pharmacokinetics of midazolam on Day 2 and Day 6.

Supplementary Figure S2: Change in HDL-C and TG levels in multiple-dose administration

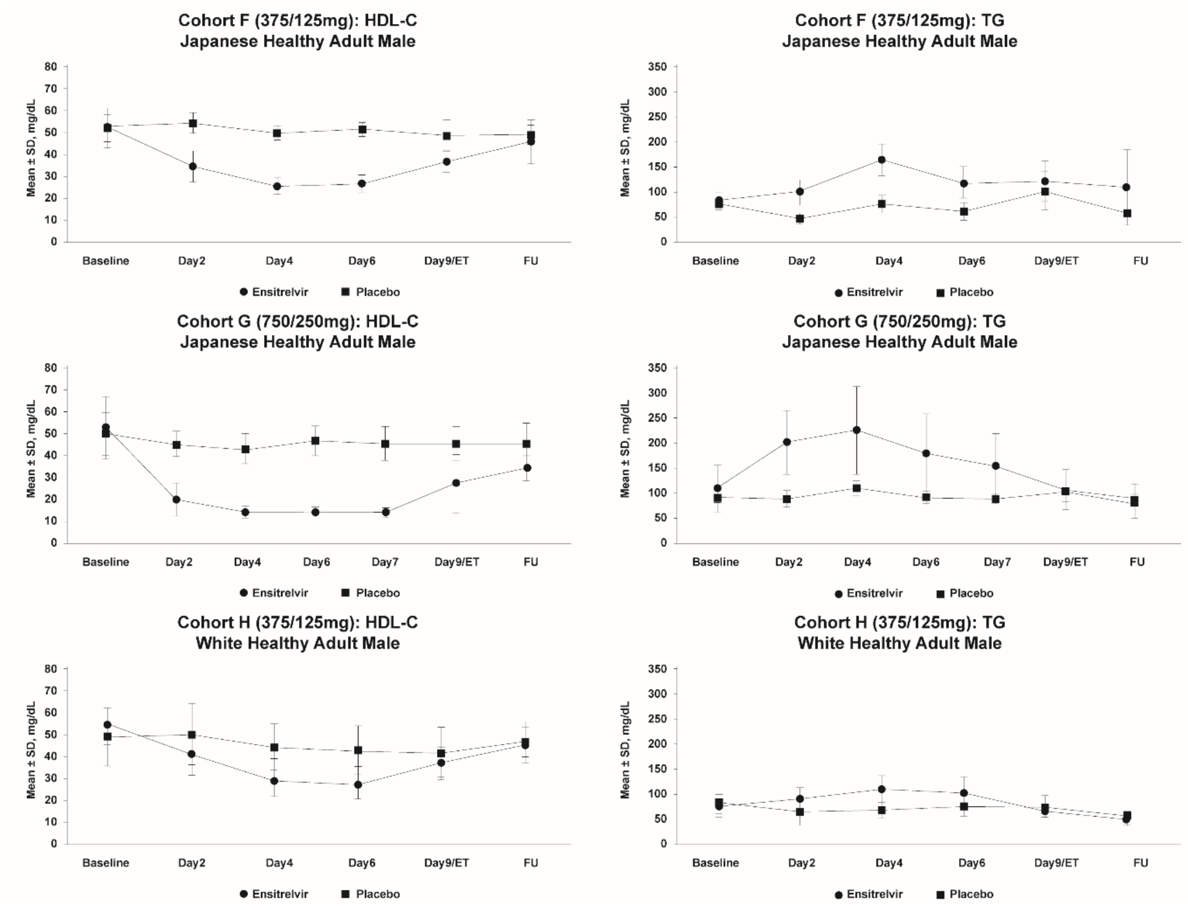

ET, early termination; FU, follow-up; HDL-C, High density lipoprotein – cholesterol; TG, triglycerides; 375/125 mg: once daily multiple dose with 375 mg as the loading dose on Day 1 and 125 mg as the maintenance dose on Days 2 to 5; 725/250 mg: once daily multiple doses with 750 mg as the loading dose on Day 1 and 250 mg as the maintenance dose on Days 2 to 5 (or 6).

Supplementary Table 1: Baseline demographics in the single dose administration part (Cohorts A, B, C, D, E, and J)

		Ensitrelvir							Placebo
		Cohort A 20 mg N = 6	Cohort B 70 mg N = 6	Cohort C1 250 mg N = 4	Cohort C2 250 mg N = 4	Cohort D 500 mg N = 6	Cohort E 1000 mg N = 6	Cohort J 2000 mg N = 6	N = 12
Sex	Male N (%)	6 (100.0)	6 (100.0)	4 (100.0)	4 (100.0)	6 (100.0)	6 (100.0)	6 (100.0)	12 (100.0)
Age (Years)	N	6	6	4	4	6	6	6	12
	Mean (SD)	28.3 (5.7)	24.0 (4.2)	27.3 (4.1)	27.5 (4.1)	22.3 (2.7)	32.8 (9.9)	29.8 (5.5)	29.3 (5.4)
	Min	20	20	22	23	20	24	23	20
	Median	28.0	22.5	28.0	27.0	21.5	30.5	31.0	29.0
	Max	36	31	31	33	26	52	37	37
Height (cm)	N	6	6	4	4	6	6	6	12
	Mean (SD)	168.98 (4.12)	172.33 (4.07)	174.78 (1.83)	172.80 (5.86)	171.98 (6.65)	171.00 (6.16)	173.82 (6.03)	170.23 (3.42)
	Min	163.7	168.3	173.3	164.3	165.4	164.2	163.6	165.0
	Median	169.95	170.95	174.20	175.10	169.10	170.20	174.10	169.95
	Max	173.0	178.7	177.4	176.7	182.5	180.5	181.6	177.1

		Ensitrelvir							Placebo
		Cohort A 20 mg N = 6	Cohort B 70 mg N = 6	Cohort C1 250 mg N = 4	Cohort C2 250 mg N = 4	Cohort D 500 mg N = 6	Cohort E 1000 mg N = 6	Cohort J 2000 mg N = 6	N = 12
Weight (kg)	N	6	6	4	4	6	6	6	12
	Mean (SD)	61.20 (5.16)	64.10 (6.11)	66.20 (5.85)	67.03 (2.52)	62.28 (7.39)	66.50 (11.15)	63.80 (4.56)	61.84 (5.05)
	Min	53.5	55.6	58.8	64.5	52.5	54.4	55.8	51.9
	Median	62.75	63.85	66.70	66.60	61.65	67.35	65.65	62.55
	Max	67.1	73.7	72.6	70.4	71.7	83.8	68.0	69.6
BMI (kg/m^2)	N	6	6	4	4	6	6	6	12
	Mean (SD)	21.33 (1.65)	21.33 (1.86)	21.30 (1.78)	22.38 (0.76)	20.75 (2.01)	22.55 (2.75)	20.83 (1.04)	21.22 (1.58)
	Min	19.2	19.0	18.8	21.7	18.9	19.5	19.3	18.8
	Median	21.40	21.00	21.80	22.20	20.10	22.10	21.00	21.55
	Max	23.8	23.9	22.8	23.4	23.9	26.1	22.0	24.0
Race	Asian N (%)	6 (100.0)	6 (100.0)	4 (100.0)	4 (100.0)	6 (100.0)	6 (100.0)	6 (100.0)	12 (100.0)
Substance usage (alcohol)	Yes	3 (50.0)	3 (50.0)	1 (25.0)	0	4 (66.7)	0	2 (33.3)	3 (25.0)
	No	3 (50.0)	3 (50.0)	3 (75.0)	4 (100.0)	2 (33.3)	6 (100.0)	4 (66.7)	9 (75.0)
Substance usage (tobacco)	Yes	0	0	0	0	0	0	0	0
	No	6 (100.0)	6 (100.0)	4 (100.0)	4 (100.0)	6 (100.0)	6 (100.0)	6 (100.0)	12 (100.0)
Substance usage (caffeine)	Yes	4 (66.7)	4 (66.7)	3 (75.0)	0	3 (50.0)	0	4 (66.7)	8 (66.7)
	No	2 (33.3)	2 (33.3)	1 (25.0)	4 (100.0)	3 (50.0)	6 (100.0)	2 (33.3)	4 (33.3)
SARS-CoV-2 test	Negative	6 (100.0)	6 (100.0)	4 (100.0)	4 (100.0)	6 (100.0)	6 (100.0)	6 (100.0)	12 (100.0)

"Placebo" includes all participants given placebo in all cohorts in Part 1. The last values taken before first administration were used if there were multiple results. Participants of Cohort C1 received administration in the fasted state on Period 1 and after a meal on Period 2. Participants of Cohort C2 received administration after a meal on Period 1 and in the fasted state on Period 2.

BMI, Body Mass Index; Max, Maximum; Min, Minimum; N, Number of participants; SARS-CoV-2, Severe Acute Respiratory Syndrome Coronavirus 2; SD, Standard Deviation.

Supplementary Table 2: Baseline demographics in the multiple-dose administration part (Cohorts F, G, and H)

		Ensitrelvir			Placebo		
		Cohort F 375/125 mg N = 8	Cohort G 750/250 mg + Midazolam N = 8	Cohort H 375/125 mg N = 8	Cohort F N = 3	Cohort G Placebo + Midazolam N = 3	Cohort H N = 3
Sex	Male N (%)	8 (100.0)	8 (100.0)	8 (100.0)	3 (100.0)	3 (100.0)	3 (100.0)
Age (Years) (informed consent obtained)	N	8	8	8	3	3	3
	Mean (SD)	33.4 (5.2)	29.4 (7.3)	36.0 (6.9)	32.3 (1.5)	30.0 (7.9)	34.0 (6.9)
	Min	25	20	26	31	21	26

		Ensitrelvir			Placebo		
		Cohort F<br>375/125 mg<br><br>N = 8	Cohort G<br>750/250 mg +<br>Midazolam<br>N = 8	Cohort H<br>375/125 mg<br><br>N = 8	Cohort F<br><br><br>N = 3	Cohort G<br>Placebo +<br>Midazolam<br>N = 3	Cohort H<br><br><br>N = 3
	Median	34.0	29.0	34.5	32.0	33.0	38.0
	Max	39	40	45	34	36	38
Height (cm)	N	8	8	8	3	3	3
	Mean (SD)	171.65 (5.12)	171.00 (8.72)	181.01 (9.58)	176.13 (1.52)	172.53 (4.39)	181.63 (7.28)
	Min	165.6	161.9	167.9	174.5	169.9	174.0
	Median	171.70	168.50	179.15	176.40	170.10	182.40
	Max	181.9	186.0	197.6	177.5	177.6	188.5
Weight (kg)	N	8	8	8	3	3	3
	Mean (SD)	66.58 (7.08)	68.03 (11.91)	74.68 (8.32)	67.03 (6.68)	62.57 (2.05)	81.13 (3.23)
	Min	60.4	55.9	62.0	60.0	60.3	78.2
	Median	63.50	65.80	75.05	67.80	63.10	80.60
	Max	80.2	85.0	87.2	73.3	64.3	84.6
BMI (kg/m^2)	N	8	8	8	3	3	3
	Mean (SD)	22.36 (1.38)	22.99 (2.54)	22.83 (2.91)	21.70 (1.70)	21.10 (1.35)	24.40 (2.00)
	Min	21.0	19.7	20.0	20.0	19.8	22.1
	Median	22.00	23.95	22.35	21.70	21.00	25.40
	Max	24.7	25.5	27.1	23.4	22.5	25.7
Race	Asian N (%)	8 (100.0)	8 (100.0)	0	3 (100.0)	3 (100.0)	0
	White N (%)	0	0	8 (100.0)	0	0	3 (100.0)
Substance usage (alcohol)	Yes	0	0	0	0	0	0
	No	8 (100.0)	8 (100.0)	8 (100.0)	3 (100.0)	3 (100.0)	3 (100.0)
Substance usage (tobacco)	Yes	0	0	0	0	0	0
	No	8 (100.0)	8 (100.0)	8 (100.0)	3 (100.0)	3 (100.0)	3 (100.0)
SARS-CoV-2 test	Negative	8 (100.0)	8 (100.0)	8 (100.0)	3 (100.0)	3 (100.0)	3 (100.0)
	Positive	0	0	0	0	0	0

The last values taken before first administration were used if there were multiple results. Participants in Cohort F and G were Japanese healthy adult males. Participants in Cohort H were White healthy adult males. 375/125 mg: once daily multiple dose with 375 mg as the loading dose on Day 1 and 125 mg as the maintenance dose on Days 2 to 5; 725/250 mg: once daily multiple doses with 750 mg as the loading dose on Day 1 and 250 mg as the maintenance dose on Days 2 to 6.

BMI, Body Mass Index; Max, Maximum; Min, Minimum; N, Number of participants; SARS-CoV-2, Severe Acute Respiratory Syndrome Coronavirus 2; SD, Standard Deviation.

Supplementary Table 3: Blood lipid profiling of participants over the study period in the single dose administration part (Cohorts A, B, C, D, E, and J)

Parameter (unit)	Time point [Cohort C, Period 2]	Statistic	Ensitrelvir Cohort A 20 mg N = 6	Cohort B 70 mg N = 6	Cohort C fasted state 250 mg N = 8	Cohort D 500 mg N = 6	Cohort E 1000 mg N = 6	Cohort J 2000 mg N = 6	Placebo N = 12
Cholesterol (mmol/L)	Baseline	n	6	6	8	6	6	6	12
		Mean (SD)	4.1635 (0.4177)	4.0987 (0.4716)	4.1053 (0.6235)	4.2838 (0.5674)	4.1332 (0.5434)	4.4608 (0.8547)	4.6333 (0.6946)
		Min	3.724	3.439	2.948	3.491	3.310	3.414	3.362
		Median	4.0730	4.0470	4.1505	4.2795	4.2410	4.2280	4.6290
		Max	4.810	4.707	4.758	5.094	4.758	5.508	5.870
	Day 2 [Day 16]	n	6	6	8	6	6	6	12
		Mean (SD)	4.2842 (0.4263)	4.1463 (0.5889)	3.8918 (0.6153)	3.9005 (0.5669)	3.5257 (0.4598)	3.9952 (0.9013)	4.7388 (0.7924)
		Min	3.905	3.388	2.870	3.155	2.767	2.870	3.414
		Median	4.1245	4.0860	3.9305	3.8660	3.6205	3.7625	4.7320
		Max	4.939	5.043	4.655	4.629	3.957	5.198	6.310
	Day 4 [Day 18]	n	6	6	8	6	6	6	12
		Mean (SD)	4.2668 (0.4032)	4.0515 (0.7018)	3.9695 (0.4928)	3.8273 (0.5428)	3.2498 (0.5322)	3.4133 (0.7824)	4.6008 (0.8016)
		Min	3.750	3.129	3.388	3.103	2.302	2.379	3.155
		Median	4.3055	3.9305	3.9825	4.0470	3.3230	3.3615	4.4735
		Max	4.810	5.017	4.577	4.293	3.905	4.422	6.413
	Day 7 [Day 21]/ Early termination	n	6	6	8	6	6	6	12
		Mean (SD)	4.1290 (0.4327)	3.8145 (0.7363)	4.0019 (0.5499)	3.8103 (0.5863)	3.7628 (0.5824)	3.9437 (0.7807)	4.4458 (0.8482)
		Min	3.646	2.974	3.103	3.129	2.664	3.077	2.948
		Median	4.1505	3.5815	4.0340	3.8535	3.9180	3.7235	4.3315
		Max	4.707	4.888	4.810	4.681	4.396	5.069	6.310
	Follow up	n	6	6	4	6	6	6	11
		Mean (SD)	3.9048 (0.2727)	3.7368 (0.6184)	3.9760 (0.7375)	3.6505 (0.4573)	3.7410 (0.5172)	3.7112 (0.6677)	4.2294 (0.7944)
		Min	3.465	2.922	2.896	2.974	2.741	3.026	3.129
		Median	3.9695	3.7110	4.2670	3.6460	3.9435	3.5430	3.9570
		Max	4.215	4.707	4.474	4.293	4.138	4.526	5.741
HDL-C (mg/L)	Baseline	n	6	6	8	6	6	6	12
		Mean (SD)	460.0 (46.0)	436.7 (46.8)	486.3 (76.1)	546.7 (127.9)	413.3 (80.7)	543.3 (82.6)	567.5 (99.8)
		Min	400	370	370	380	300	420	450
		Median	470.0	445.0	510.0	540.0	425.0	535.0	530.0
		Max	510	480	570	710	530	640	750
	Day 2 [Day 16]	n	6	6	8	6	6	6	12
		Mean (SD)	458.3 (47.9)	413.3 (42.7)	376.3 (81.9)	330.0 (106.6)	166.7 (45.5)	225.0 (76.6)	569.2 (99.6)
		Min	390	360	220	200	100	150	450
		Median	465.0	430.0	380.0	325.0	175.0	200.0	530.0
		Max	520	450	470	460	220	330	770
	Day 4 [Day 18]	n	6	6	8	6	6	6	12
		Mean (SD)	446.7 (63.1)	411.7 (52.3)	401.3 (66.4)	340.0 (74.8)	180.0 (52.5)	171.7 (55.6)	538.3 (94.9)
		Min	370	340	300	230	140	120	440
		Median	450.0	420.0	390.0	340.0	165.0	155.0	510.0
		Max	510	470	480	440	280	280	730
	Day 7 [Day 21]/ Early termination	n	6	6	8	6	6	6	12
		Mean (SD)	440.0 (66.9)	396.7 (60.2)	438.8 (76.6)	421.7 (83.8)	303.3 (61.5)	253.3 (105.0)	531.7 (107.7)

		Min	370	320	330	280	210	40	410
		Median	445.0	400.0	445.0	440.0	305.0	295.0	505.0
		Max	510	480	530	500	400	310	750
	Follow up	n	6	6	4	6	6	6	11
		Mean (SD)	445.0 (70.1)	420.0 (55.1)	445.0 (113.9)	450.0 (85.1)	343.3 (47.2)	405.0 (45.9)	533.6 (113.2)
		Min	370	350	340	320	260	360	410
		Median	440.0	435.0	425.0	470.0	350.0	395.0	500.0
		Max	530	490	590	540	400	490	760
LDL-C (mg/L)	Baseline	n	6	8	8	6	6	6	12
		Mean (SD)	993.3 (142.4)	995.0 (194.6)	900.0 (199.3)	990.0 (228.7)	986.7 (175.3)	1051.7 (296.1)	1052.5 (263.9)
		Min	850	770	540	710	690	750	630
		Median	970.0	920.0	865.0	945.0	1015.0	995.0	1020.0
		Max	1230	1310	1150	1360	1170	1410	1620
	Day 2 [Day 16]	n	6	6	8	6	6	6	12
		Mean (SD)	1048.3 (164.1)	1041.7 (241.5)	916.3 (179.2)	951.7 (219.6)	840.0 (141.8)	901.7 (268.8)	1112.5 (317.2)
		Min	900	790	580	700	580	630	610
		Median	1015.0	965.0	885.0	925.0	880.0	815.0	1075.0
		Max	1310	1470	1150	1250	970	1250	1910
	Day 4 [Day 18]	n	6	6	8	6	6	6	12
		Mean (SD)	1026.7 (152.3)	946.7 (254.0)	942.5 (140.8)	950.0 (219.1)	800.0 (159.1)	865.0 (239.7)	1047.5 (320.9)
		Min	840	670	740	620	540	550	550
		Median	1050.0	880.0	930.0	985.0	875.0	870.0	1000.0
		Max	1190	1360	1160	1200	940	1200	1900
	Day 7 [Day 21]/ Early termination	n	6	6	8	6	6	6	12
		Mean (SD)	966.7 (150.6)	881.7 (293.5)	923.8 (136.4)	928.3 (263.5)	1003.3 (198.3)	1135.0 (327.0)	1015.8 (346.6)
		Min	770	620	710	610	620	820	500
		Median	1015.0	790.0	900.0	900.0	1080.0	1020.0	955.0
		Max	1110	1370	1130	1350	1160	1610	1900
	Follow up	n	6	6	4	6	6	6	11
		Mean (SD)	923.3 (104.6)	873.3 (242.9)	977.5 (213.0)	843.3 (216.1)	988.3 (155.1)	951.7 (249.0)	956.4 (299.2)
		Min	790	630	680	630	680	700	580
		Median	915.0	805.0	1030.0	795.0	1035.0	865.0	920.0
		Max	1080	1320	1170	1170	1100	1260	1490
Triglyceride (mmol/L)	Baseline	n	6	6	8	6	6	6	12
		Mean (SD)	0.8825 (0.2807)	1.0330 (0.3537)	0.9596 (0.4428)	0.7507 (0.1445)	1.0275 (0.3223)	0.9975 (0.4064)	0.8702 (0.3224)
		Min	0.644	0.485	0.474	0.632	0.565	0.542	0.440
		Median	0.7730	1.0615	0.8245	0.7055	1.0840	1.0220	0.7730
		Max	1.321	1.479	1.863	1.016	1.332	1.479	1.671
	Day 2 [Day 16]	n	6	6	8	6	6	6	12
		Mean (SD)	1.0032 (0.2053)	1.1478 (0.2935)	1.3053 (0.6212)	1.2928 (0.2879)	2.3427 (0.6155)	2.0737 (0.5253)	0.9314 (0.2873)
		Min	0.700	0.711	0.598	1.039	1.456	1.524	0.463
		Median	1.0165	1.0895	1.1345	1.1740	2.2295	1.9475	0.8525
		Max	1.310	1.547	2.506	1.818	3.105	2.789	1.603
	Day 4 [Day 18]	n	6	6	8	6	6	6	12

	Mean (SD)	1.3643 (0.2213)	1.4170 (0.5431)	1.2235 (0.3569)	1.1327 (0.1334)	1.9683 (0.5250)	1.7668 (0.4684)	1.2945 (0.4778)
	Min	1.084	0.948	0.892	0.937	1.321	1.073	0.745
	Median	1.2985	1.3270	1.0670	1.1400	1.8855	1.8120	1.1400
	Max	1.671	2.427	1.795	1.276	2.755	2.303	2.427
Day 7 [Day 21]/ Early termination	n	6	6	8	6	6	6	12
	Mean (SD)	1.3852 (0.2059)	1.4393 (0.6236)	1.0571 (0.4382)	0.9937 (0.1403)	1.2210 (0.5146)	0.7867 (0.1677)	1.1950 (0.3927)
	Min	1.039	0.858	0.790	0.779	0.632	0.508	0.655
	Median	1.4285	1.2810	0.8470	0.9770	1.1570	0.8130	1.1745
	Max	1.581	2.608	2.010	1.174	1.818	0.971	1.897
Follow up	n	6	6	4	6	6	6	11
	Mean (SD)	0.7037 (0.1371)	0.8882 (0.4558)	0.6295 (0.1096)	0.5627 (0.0611)	0.7565 (0.3775)	0.4255 (0.1538)	0.8015 (0.7116)
	Min	0.587	0.305	0.542	0.463	0.463	0.294	0.327
	Median	0.6605	0.7620	0.5985	0.5700	0.5480	0.3615	0.5870
	Max	0.948	1.626	0.779	0.632	1.264	0.689	2.845

"Placebo" includes all participants given placebo in all cohorts in Part 1. The last values taken before first administration were used if there were multiple results. Four participants of Cohort C received administration in the fasted state on Period 1 and after a meal on Period 2. The other four participants of Cohort C received administration after meal on Period 1 and in the fasted state on Period 2 (Day 16, 18 and 21).

HDL-C, high density lipoprotein-Cholesterol; LDL-C, low density lipoprotein-Cholesterol; Max, Maximum; Min, Minimum; N, Number of participants; SD, Standard Deviation.

Supplementary Table 4: Study design

(A) Single-dose administration

Cohort		Treatment	Sample size	Total Sample size	Food condition	Participants
A	A1	Ensitrelvir 20 mg	1	8	Fasted	Japanese healthy adult male
		Placebo	1			
	A2	Ensitrelvir 20 mg	5			
		Placebo	1			
B		Ensitrelvir 70 mg	6	8		
		Placebo	2			
C[a]	C1	Ensitrelvir 250 mg	4	5	Fasted in Period 1 and Fed[b] in Period 2	
		Placebo	1			
	C2	Ensitrelvir 250 mg	4	5	Fed[b] in Period 1 and Fasted in Period 2	
		Placebo	1			
D		Ensitrelvir 500 mg	6	8	Fasted	
		Placebo	2			
E		Ensitrelvir 1000 mg	6	8		

		Placebo	2	
J	J1	Ensitrelvir 2000 mg	1	8
		Placebo	1	
	J2	Ensitrelvir 2000 mg	5	
		Placebo	1	

[a]The effect of food was assessed in Cohort C; [b]High-fat/high-calorie meal, after breakfast.

(B) Multiple-dose administration

Cohort	Treatment: Loading dose (Day 1)	Treatment: Maintenance dose (Days 2-5/6)	Group	Sample size	Total sample size	Participants
F	375 mg	125 mg	Ensitrelvir	8	11	Japanese healthy adult male
			Placebo	3		
G[a]	750 mg	250 mg	Ensitrelvir	8	11	
			Placebo	3		
H	375 mg	125 mg	Ensitrelvir	8	11	White healthy adult male
			Placebo	3		

[a]The effect of multiple-dose administration of ensiltrelvir on the pharmacokinetics of midazolam (single dose 2mg [1 mL syrup]) was investigated.
